# The behavioral effects of antibiotic treatment on the snail *Biomphalaria glabrata*

**DOI:** 10.7717/peerj.4171

**Published:** 2017-12-21

**Authors:** Euan R.O. Allan, Michael S. Blouin

**Affiliations:** Integrative Biology, Oregon State University, Corvallis, OR, United States of America

**Keywords:** *Biomphalaria glabrata*, Schistosome, Antibiotics, Activity

## Abstract

Schistosomiasis is a detrimental neglected tropical disease that is transmitted by Planorbid snails. Understanding the transmission and control of this disease requires an extensive understanding of these intermediate hosts, which is only achieved by the effective rearing and study of species such as *Biomphalaria glabrata*. This species is the intermediate host for *Schistosoma mansoni* in the New World, and is also the main model for studying schistosomes in mollusks. Antibiotics are used routinely in *B. glabrata* tissue culture, and occasionally on live snails. Here we show that standard doses of three common antibiotics (penicillin, streptomycin and gentamicin) drastically diminish the activity of healthy *B. glabrata*, but that treated snails recover rapidly when placed in fresh water. Ampicillin treated snails did not show altered activity. We suggest that researchers keep these apparent toxicities in mind if a need for antibiotic treatment of live Planorbid snails arises.

## Introduction

Planorbid snails transmit numerous mammalian parasites including: lungworms and liver, intestinal, and blood flukes (Giannelli et al., 2016; Loker, 2010). These snails act as intermediate molluscan hosts, and are essential for perpetuating diseases which detrimentally affect the health of both humans and livestock (Pearce & MacDonald, 2002; Sokolow et al., 2016). In an effort to understand and control the spread of snail-borne parasitic diseases, extensive resources have been expended in developing models of parasitic infection in planorbid snails, particularly in the species *Biomphalaria glabrata,* with the aim of understanding and blocking the transmission of parasites to humans. This species is primarily responsible for transmission of the human pathogen *Schistosoma mansoni* in the Americas and, for this reason, has been under intense experimental scrutiny for decades (Allan et al., 2017; Pearce & MacDonald, 2002; Reardon, 2016).

Successful husbandry practices for *B. glabrata* are well established, and are relatively simple in comparison to other vertebrate model organisms (Ducklow et al., 1979; Galinier et al., 2017; Hanington et al., 2010; Jiang, Loker & Zhang, 2006). These practices do not generally involve the use of antibiotics, as a heathy microbial population is important in many aquatic environments, and these snails are not kept in a germ-free environment (Chernin, 1957; Chernin & Schork, 1959). Despite their rare use during snail husbandry, standard doses of penicillin/streptomycin (P/S) are commonly used to culture the snail 1818 embryonic Bge cell line as well as isolated hemocytes from the snail hemolymph, and have been used on whole snails in the past (Bender et al., 2005; Chernin, 1957; Chernin & Schork, 1959; Chernin & Schork, 1960; Goodall et al., 2006; Hahn, Bender & Bayne, 2000; Hahn, Bender & Bayne, 2001a; Hahn, Bender & Bayne, 2001b; Yoshino, Bickham & Bayne, 2013). Snails treated with streptomycin have been shown to have modified growth as hatchlings, but antibiotic use has not caused any reported cellular dysregulation in culture (Chernin & Schork, 1960). Here we report that several commonly-used antibiotics have very strong behavioral effects on adult *B. glabrata.*

This study was motivated by an unexpected and anecdotal observation of apparent intoxication in snails treated with P/S. When a number of tanks of snails became contaminated with a putative bacterium, we treated one tank with P/S and observed drastic behavioral changes in our adult snails. This prompted us to examine the effects of four common antibiotics and a commonly used antibiotic cocktail on the activity of healthy *B. glabrata* in uncontaminated conditions. We show that three commonly-used antibiotics may have detrimental physiological effects on *B. glabrata*, resulting in drastic changes in the level of activity of exposed snails, and thus should be carefully trialed before use on experimental snails (e.g., for decontamination or microbiome interrogation). This report should serve as a stepping stone for the future study of the effects of antibiotics on *B. glabrata*, and as a reminder that this species is vulnerable to uncharacterized toxic effects of some of these compounds.

## Materials and Methods

Guadeloupean *B. glabrata* (BgGUA) was collected in 2005 from the island of Guadeloupe, and maintained under standard conditions as previously described (Tennessen et al., 2015; Theron et al., 2008; Theron et al., 2014). Snails were housed, fed identically, and size matched (6–8 mm). All reagents were acquired from Sigma-Aldrich and used according to the manufactures instructions (suggested doses).

Initial anecdotal observation: During normal husbandry of BgGUA, we found multiple tanks full of foul-smelling flocculent brown masses, which we had never previously observed, less than a week after cleaning. High adult snail mortality (>70%) was observed in these tanks. When water from a contaminated tank (100 ml) was removed, placed into two small vessels, and treated with P/S; the brown flocculence dissipated within two days in the antibiotic treated vessel, while the other vessel remained unchanged. We postulated that there was some bacterial contaminant from our source water, as this only occurred in tanks using a single water source. Tanks that received water from a different source appeared normal. Thus, a single contaminated tank was treated with P/S, and within three days the water was clear (no abnormal biofilms or brown flocculence) but the snails appeared to be dead so antibiotics were not used on any additional tanks. This tank (and everything in it) was then bleached extensively, to avoid the spread of any surviving fouling organism, and the water was autoclaved and bleached to ensure that no resistant bacteria or antibiotics were released into the environment. The remaining contamination was overcome by individually cleaning each rescued snail’s surface with 70% ethanol, and moving them to a freshly cleaned tank with water from a different source (no use of antibiotics). This was repeated every week for a month and no subsequent contamination was observed. The microbial contaminant, putatively a bacterium, was never positivity identified.

To examine the effects of different antibiotic treatments on BgGUA, we exposed snails (from uncontaminated tanks) to increasing doses of P/S (10,000 U penicillin, 10 mg/ml streptomycin, suggested effective dose 1/100) from 1/1000 to 1/100 (1/100 is the suggested dose for bacterial control) for a 24 h period. Additionally, we used common doses (manufacturer’s instructions; Sigma-Aldrich, St. Louis, MO, USA) of P/S (100 U/ml penicillin with 10 mg/L streptomycin: equivalent to 1/100 suggested dose), penicillin G (100 U/ml, in water), streptomycin sulfate (100 mg/L, in water), gentamicin sulfate (50 mg/L, in water), and ampicillin (100 mg/L, in water). All of the snails used in these experiments originated from uncontaminated tanks, and were kept in fresh water. Doses of antibiotics are equivalent to those used previously on whole *B. glabrata*, as well as those used in some aquaculture settings (Chernin & Schork, 1959; Chernin & Schork, 1960; Gilmartin, Camp & Lewis, 1976). We did not measure the tissue concentration of each antibiotic in whole snails. All control snails were with sodium citrate (1 mM) alone. All antibiotic treated water/tanks were autoclaved and bleached after use. Each group of treated and control snails was housed in 1 L of fresh (uncontaminated) water, in separate four L non-reactive plastic tanks (∼100−160 ml/snail), and each treatment was repeated on three independent occasions (*n* = 3 experiments each with, 6–10 snails/treatment). The temperature and pH were verified daily (pH = 7 +∕ − 0.2, Temperature = 26 °C +∕ − 0.4 °C). We counted the number of active snails at 15 min, 30 min, 45 min, 1 h, 2 h, 4 h, 24 h, 48 h, and 72 h after being dosed with antibiotics. Snails were placed in fresh tanks every 24 h to maintain a relatively consistent antibiotic concentration as the stability of these antibiotics begins to decrease after 48 h at 37 °C (Sigma manufacturer’s instructions). Although the stabilities of these antibiotics differ in aqueous solution, all four have been shown to maintain efficacy aver a 24 h period at temperatures below 30 °C (Benedict, Schmidt & Coghill, 1946; Cote et al., 2010; Fujiwara, Kawashima & Ohhashi, 1982; Macek, Hanus & Feller, 1948; Oswald & Nielsen, 1947; Pang, Guan & Cheng, 1984; Schwartz & Hayton, 1972). Each time snails were moved they were given fresh food (lettuce). Control snails consumed food normally while treated snails consumed no food. After 72 h we rescued all the snails into untreated water, and examined their activity after 73 h (1 h post rescue), and 96 h. We considered a snail to be active if it was moving, feeding, mating, or attached to a surface by its headfoot. Inactive snails were lying on their sides on the bottom of the tank, motionless, or fully withdrawn into their shell. Additionally, these inactive snails did not respond to mechanical stimuli (a gentle prodding with soft tweezers), and appeared to be moribund. There was no mortality of snails in any treatment. The EC50 for P/S was calculated using a variable slope model, (*Y* = Bottom  + (Top−Bottom))/(1 + 10ˆ((LogEC50−*X*)^∗^HillSlope)). Statistical analyses of activity were completed by One-way ANOVA with a Tukey’s post-hoc test (*p* < 0.05). All analyses were completed using GraphPad Prism software (La Jolla, CA, USA).

## Results

**Figure 1:**
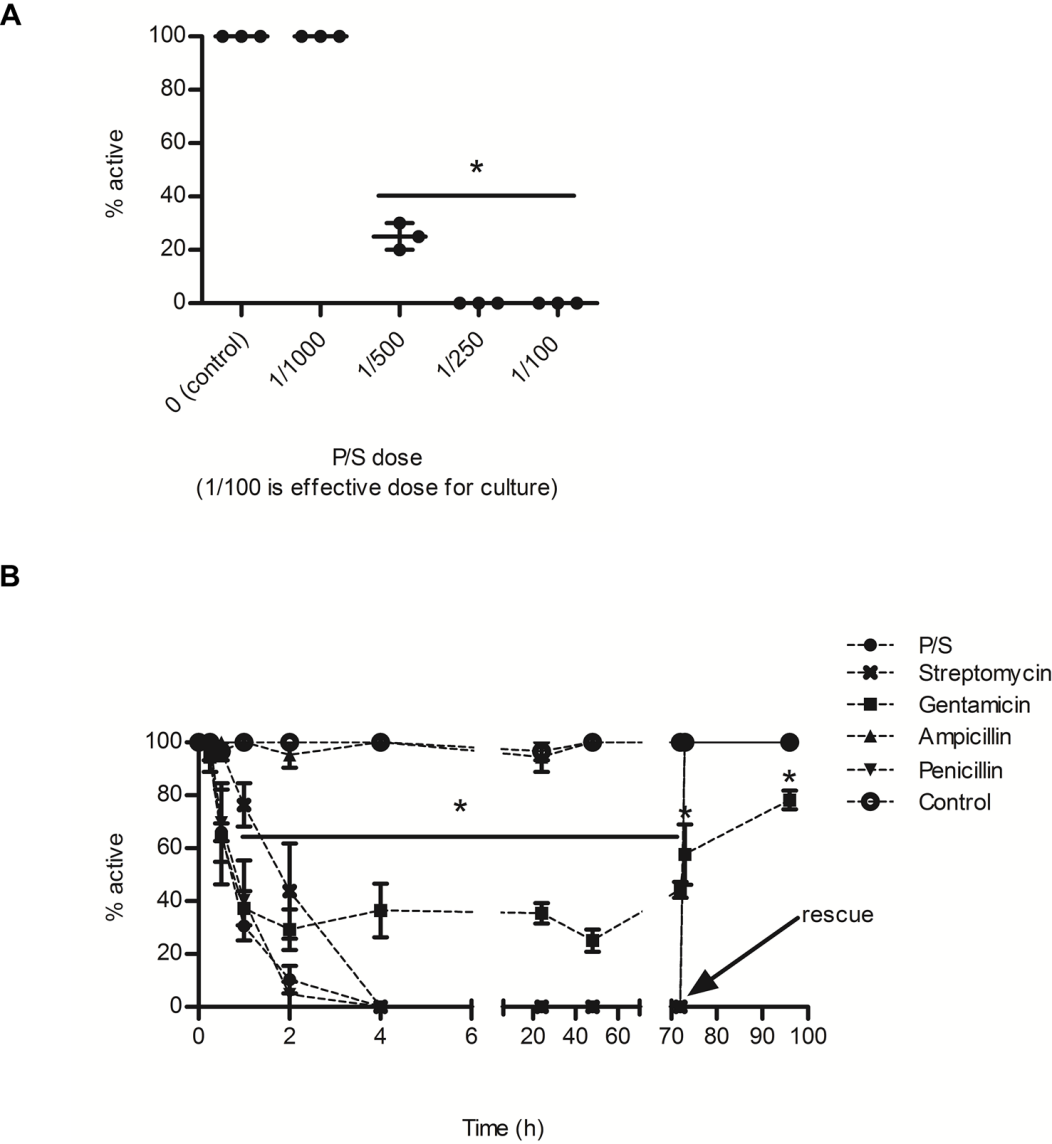
The effects of common antibiotics on the activity of BgGUA. (A) The percent of active BgGUA 24 h after dosing with 0/1000, 1/1000, 1/500, 1/250, and 1/100 penicillin/streptomycin (P/S) (*n* = 3, 6–10 snails per experiment). (B) The percent of active BgGUA over 72 h with standard doses of 4 individual antibiotics and one combination (P/S) treatment (*n* = 3, 6–10 snails per experiment). Control snails were treated with sodium citrate buffer alone. All snails were rescued to untreated water after 72 h and activity was monitored for an additional 24 h. The EC50 was ∼1/450. Data are presented as mean % active snails +∕ − standard deviation. Significant differences from (A) 0/1000 or the (B) control (One-way ANOVA, Tukey’s post-hoc, *p* < 0.05) in are denoted by an asterisks (*).

P/S significantly reduced the percentage of active BgGUA snails (Fig. 1A). 80% of snails were inactive when exposed to P/S doses as low as 5 fold less than suggested doses for bacterial suppression, and the EC50 for P/S was ∼1/450 (18 U/L of penicillin, 0.18 mg/L streptomycin) (Fig. 1A). This inactivity was also observed when examined over a 96 h time-course with three individual antibiotics. Penicillin G and streptomycin completely ablated snail activity within the first 2 h of exposure, while gentamicin reduced snail activity to less than 40% of the control (Fig. 1B). Interestingly, ampicillin, a penicillin derivative, did not alter activity (Acred et al., 1962). When snails were rescued, in fresh water after three days of antibiotic exposure, they recovered within the first hour (Fig. 1B). Gentamicin treated snails did not fully recover and remained statistically less active than all other rescued snails (Fig. 1B).

## Discussion and Conclusions

Our findings indicate that antibiotic induced intoxication is both rapid in onset, and reversible in most cases. We do not believe that the effects these antibiotics had on the behavior of the snails were the result of modifications to the bacterial microenvironment of the tank or the animal, because of the extremely rapid onset of inactivity and rapid recovery of activity. Although P/S has been consistently and successfully used to maintain sterile cultures of snail derived cells, these data suggest it requires additional characterization before it is used to treat adult planorbid snails. We did not show a reduction in activity of ampicillin exposed snails, so it is possible that ampicillin could be used as a broad spectrum replacement for P/S with *B. glabrata*, but a more complete study and dose response should be done before it is used in snail husbandry. Gentamycin-treated snails exhibited a milder phenotype, but did not recover as quickly when they were rescued. Other anti-microbial pharmacologics have been shown to negatively affect *B. glabrata* but no study, to our knowledge, has shown that these common antibiotics disrupt snail behavior (Katz et al., 2017). Interestingly, some antibiotics can have neurotoxic effects in mammals, and it is possible that BgGUA experience some neurotoxic effects when exposed to standard doses of these antibiotics (Grill & Maganti, 2011). Additionally, it has been previously reported that similar doses of streptomycin can inhibit the growth of *B. glabrata* hatchlings (Chernin, 1957; Chernin & Schork, 1959; Chernin & Schork, 1960). We add to these initial studies, and show that the toxic effects of streptomycin in adult snails can be acute and behavioral. They are also similar to the distress syndrome that can occur in snails after they are exposed to extreme ionic conditions, severely elevated carbon dioxide, and heavy metals (Harry, 1967; Yager & Harry, 1966). Our observations are not predicted by the mechanism of anti-bacterial action of each of these antibiotics, so it would be difficult to predict how *B. glabrata* would react to antibiotics that were not examined in this small study. A more extensive characterization of the physiological effects of all of these antibiotics, including ampicillin, should be done. This future work should also examine the potential mechanism behind these phenomena, determine if other concentrations or alternative compounds are less detrimental to snail behavior, or if changes can be made to the environment to reduce the toxicity of these compounds (i.e., pH). It would also be prudent to examine snail tissue, and determine if the internal concentrations of these antibiotics differ, as bioaccumulation could play a role in toxicity (Hoke et al., 2016).

The study of planorbid snail physiology and immunity is important for fully understanding parasitic worm infections, and could be critical for controlling the extent of these mammalian diseases. Additionally, model organisms, such as *B. glabrata*, have provided essential insights into molluscan physiology and immunology. Healthy and controlled husbandry of planorbid snails is vital for the observations of repeatable biological phenotypes, and being aware of detrimental practices is crucial for promoting consistencies between institutions. In light of the recent advances in the study of the microbiome of different organisms, it is important to understand which antibiotics could be used to modify the microbiome of snails without having severe behavioral effects (Rooks & Garrett, 2016; Spor, Koren & Ley, 2011; Thaiss et al., 2016). Understanding which antibiotics could be used to generate relatively “germ free” snails, with the goal of examining the roles of the microbiome in snail physiology or defense, is essential given that efficient axenic culture of *B. glabrata* does not permit normal growth and feeding (Chernin & Schork, 1959). In summary, the present study reports that common antibiotics can have severe effects on the behavioral activity, and potentially the health, of *B. glabrata*, and should be thoroughly examined by researchers before extensive use on experimental animals.

##  Supplemental Information

10.7717/peerj.4171/supp-1 Data S1 Raw data Two tables with the corresponding raw data for Figs. 1A and 1B.

## Additional Information and Declarations

### Data Availability

The following information was supplied regarding data availability: The raw data has been provided as a Supplemental File.

## References

[ref-1] Acred P, Brown DM, Turner DH, Wilson MJ (1962). Pharmacology and chemotherapy of ampicillin—a new broad-spectrum penicillin. British Journal of Pharmacology and Chemotherapy.

[ref-2] Allan ER, Tennessen JA, Bollmann SR, Hanington PC, Bayne CJ, Blouin MS (2017). Schistosome infectivity in the snail, *Biomphalaria glabrata*, is partially dependent on the expression of Grctm6, a Guadeloupe Resistance Complex protein. PLOS Neglected Tropical Diseases.

[ref-3] Bender RC, Broderick EJ, Goodall CP, Bayne CJ (2005). Respiratory burst of *Biomphalaria glabrata* hemocytes: *Schistosoma mansoni*-resistant snails produce more extracellular H2O2 than susceptible snails. Journal of Parasitology.

[ref-4] Benedict RG, Schmidt WH, Coghill RD (1946). The stability of penicillin in aqueous solution. Journal of Bacteriology.

[ref-5] Chernin E (1957). A method of securing bacteriologically sterile snails (Australorbis glabratus). Proceedings of the Society for Experimental Biology and Medicine.

[ref-6] Chernin E, Schork AR (1959). Growth in axenic culture of the snail, Australorbis glabratus. American Journal of Hygiene.

[ref-7] Chernin E, Schork AR (1960). Effects of streptomycin on the hatching of Australorbis glabratus eggs. Experimental Parasitology.

[ref-8] Cote D, Lok CE, Battistella M, Vercaigne L (2010). Stability of trisodium citrate and gentamicin solution for catheter locks after storage in plastic syringes at room temperature. Canadian Journal of Hospital Pharmacy.

[ref-9] Ducklow HW, Boyle PJ, Maugel PW, Strong C, Mitchell R (1979). Bacterial flora of the schistosome vector snail *Biomphalaria glabrata*. Applied and Environmental Microbiology.

[ref-10] Fujiwara H, Kawashima S, Ohhashi M (1982). Stabilization of ampicillin analogs in aqueous solution. I. Assay of Ampicillin in solutions containing benzaldehyde by iodine colorimetry and the effect of benzaldehyde on the stability of ampicillin. Chemical and Pharmaceutical Bulletin.

[ref-11] Galinier R, Tetreau G, Portet A, Pinaud S, Duval D, Gourbal B (2017). First characterization of viruses from freshwater snails of the genus *Biomphalaria*, the intermediate host of the parasite *Schistosoma mansoni*. Acta Tropica.

[ref-12] Giannelli A, Cantacessi C, Colella V, Dantas-Torres F, Otranto D (2016). Gastropod-Borne Helminths: a look at the snail-parasite interplay. Trends in Parasitology.

[ref-13] Gilmartin WG, Camp BJ, Lewis DH (1976). Bath treatment of channel catfish with three broad-spectrum antibiotics. Journal of Wildlife Diseases.

[ref-14] Goodall CP, Bender RC, Brooks JK, Bayne CJ (2006). *Biomphalaria glabrata* cytosolic copper/zinc superoxide dismutase (SOD1) gene: association of SOD1 alleles with resistance/susceptibility to *Schistosoma mansoni*. Molecular and Biochemical Parasitology.

[ref-15] Grill MF, Maganti RK (2011). Neurotoxic effects associated with antibiotic use: management considerations. British Journal of Clinical Pharmacology.

[ref-16] Hahn UK, Bender RC, Bayne CJ (2000). Production of reactive oxygen species by hemocytes of *Biomphalaria glabrata*: carbohydrate-specific stimulation. Developmental and Comparative Immunology.

[ref-17] Hahn UK, Bender RC, Bayne CJ (2001a). Involvement of nitric oxide in killing of *Schistosoma mansoni* sporocysts by hemocytes from resistant *Biomphalaria glabrata*. Journal of Parasitology.

[ref-18] Hahn UK, Bender RC, Bayne CJ (2001b). Killing of *Schistosoma mansoni* sporocysts by hemocytes from resistant *Biomphalaria glabrata*: role of reactive oxygen species. Journal of Parasitology.

[ref-19] Hanington PC, Forys MA, Dragoo JW, Zhang SM, Adema CM, Loker ES (2010). Role for a somatically diversified lectin in resistance of an invertebrate to parasite infection. Proceedings of the National Academy of Sciences of the United States of America.

[ref-20] Harry HW (1967). Tolerance of snail taphius glabratus (Say) to major ions which occur in freshwaters. Texas Journal of Science.

[ref-21] Hoke R, Huggett D, Brasfield S, Brown B, Embry M, Fairbrother A, Kivi M, Paumen ML, Prosser R, Salvito D, Scroggins R (2016). Review of laboratory-based terrestrial bioaccumulation assessment approaches for organic chemicals: current status and future possibilities. Integrated Environmental Assessment and Management.

[ref-22] Jiang Y, Loker ES, Zhang SM (2006). *In vivo* and *in vitro* knockdown of FREP2 gene expression in the snail *Biomphalaria glabrata* using RNA interference. Developmental and Comparative Immunology.

[ref-23] Katz N, Araujo N, Coelho PMZ, Morel CM, Linde-Arias AR, Yamada T, Horimatsu Y, Suzuki K, Sunazuka T, Omura S (2017). Ivermectin efficacy against *Biomphalaria*, intermediate host snail vectors of Schistosomiasis. Journal of Antibiotics.

[ref-24] Loker ES (2010). Gastropod immunobiology. Advances in Experimental Medicine and Biology.

[ref-25] Macek TJ, Hanus EJ, Feller BA (1948). The stability of penicillin G sodium in aqueous solution. Journal of the American Pharmacists Association.

[ref-26] Oswald EJ, Nielsen JK (1947). Studies on the stability of streptomycin in solution. Science.

[ref-27] Pang YH, Guan KL, Cheng AP (1984). Study on chemical stability of streptomycin sulfate in an aqueous solution. Yao Xue Xue Bao.

[ref-28] Pearce EJ, MacDonald AS (2002). The immunobiology of schistosomiasis. Nature Reviews Immunology.

[ref-29] Reardon S (2016). Welcome to the CRISPR zoo. Nature.

[ref-30] Rooks MG, Garrett WS (2016). Gut microbiota, metabolites and host immunity. Nature Reviews Immunology.

[ref-31] Schwartz MA, Hayton WL (1972). Relative stability of hetacillin and ampicillin in solution. Journal of Pharmaceutical Sciences.

[ref-32] Sokolow SH, Wood CL, Jones IJ, Swartz SJ, Lopez M, Hsieh MH, Lafferty KD, Kuris AM, Rickards C, De Leo GA (2016). Global assessment of schistosomiasis control over the past century shows targeting the snail intermediate host works best. PLOS Neglected Tropical Diseases.

[ref-33] Spor A, Koren O, Ley R (2011). Unravelling the effects of the environment and host genotype on the gut microbiome. Nature Reviews Microbiology.

[ref-34] Tennessen JA, Theron A, Marine M, Yeh JY, Rognon A, Blouin MS (2015). Hyperdiverse gene cluster in snail host conveys resistance to human schistosome parasites. PLOS Genetics.

[ref-35] Thaiss CA, Zmora N, Levy M, Elinav E (2016). The microbiome and innate immunity. Nature.

[ref-36] Theron A, Coustau C, Rognon A, Gourbiere S, Blouin MS (2008). Effects of laboratory culture on compatibility between snails and schistosomes. Parasitology.

[ref-37] Theron A, Rognon A, Gourbal B, Mitta G (2014). Multi-parasite host susceptibility and multi-host parasite infectivity: a new approach of the *Biomphalaria glabrata/Schistosoma mansoni* compatibility polymorphism. Infection, Genetics and Evolution.

[ref-38] Yager CM, Harry HW (1966). Uptake of heavy metal ions by Taphius glabratus, a snail host of *Schistosoma mansoni*. Experimental Parasitology.

[ref-39] Yoshino TP, Bickham U, Bayne CJ (2013). Molluscan cells in culture: primary cell cultures and cell lines. Canadian Journal of Zoology.

